# Variation in Community Structure across Vertical Intertidal Stress Gradients: How Does It Compare with Horizontal Variation at Different Scales?

**DOI:** 10.1371/journal.pone.0024062

**Published:** 2011-08-24

**Authors:** Nelson Valdivia, Ricardo A. Scrosati, Markus Molis, Amanda S. Knox

**Affiliations:** 1 Instituto de Ciencias Marinas y Limnológicas, Facultad de Ciencias, Universidad Austral de Chile, Valdivia, Chile; 2 Centro de Estudios Avanzados de Zonas Áridas (CEAZA), Facultad de Ciencias del Mar, Universidad Católica del Norte, Coquimbo, Chile; 3 Saint Francis Xavier University, Department of Biology, Antigonish, Nova Scotia, Canada; 4 Section Functional Ecology, Biologische Anstalt Helgoland, Alfred Wegener Institute for Polar and Marine Research, Helgoland, Germany; National Institute of Water & Atmospheric Research, New Zealand

## Abstract

In rocky intertidal habitats, the pronounced increase in environmental stress from low to high elevations greatly affects community structure, that is, the combined measure of species identity and their relative abundance. Recent studies have shown that ecological variation also occurs along the coastline at a variety of spatial scales. Little is known, however, on how vertical variation compares with horizontal variation measured at increasing spatial scales (in terms of sampling interval). Because broad-scale processes can generate geographical patterns in community structure, we tested the hypothesis that vertical ecological variation is higher than fine-scale horizontal variation but lower than broad-scale horizontal variation. To test this prediction, we compared the variation in community structure across intertidal elevations on rocky shores of Helgoland Island with independent estimates of horizontal variation measured at the scale of patches (quadrats separated by 10s of cm), sites (quadrats separated by a few m), and shores (quadrats separated by 100s to 1000s of m). The multivariate analyses done on community structure supported our prediction. Specifically, vertical variation was significantly higher than patch- and site-scale horizontal variation but lower than shore-scale horizontal variation. Similar patterns were found for the variation in abundance of foundation taxa such as *Fucus* spp. and *Mastocarpus stellatus*, suggesting that the effects of these canopy-forming algae, known to function as ecosystem engineers, may explain part of the observed variability in community structure. Our findings suggest that broad-scale processes affecting species performance increase ecological variability relative to the pervasive fine-scale patchiness already described for marine coasts and the well known variation caused by vertical stress gradients. Our results also indicate that experimental research aiming to understand community structure on marine shores should benefit from applying a multi-scale approach.

## Introduction

During the last several years, it has become evident that the different processes that affect species distribution operate and generate variability at different spatial scales [1], [2]. Thus, ecological research has evolved from considering spatial variation in species assemblages as “noise” to appreciating that, in fact, identifying spatial scales of ecological variation is important to understanding how species assemblages are structured. Studies focused on determining spatial scales of variation have been especially prolific for rocky intertidal systems, for which patterns in community structure have indeed been shown to depend on the scale of sampling [3]–[5].

The rocky intertidal habitat is a unique system because a strong environmental stress gradient occurs within a few metres from low to high elevations [6]. The regular alternation of high and low tides determines that the duration of exposure to the air increases with elevation. Because of that, the abiotic stresses related to temperature, desiccation, irradiance, and osmotic potential increase towards the high intertidal zone, where they exhibit their most extreme values [7], [8]. Ubiquitous patterns of species distribution along this vertical stress gradient (vertical variation) have inspired an extensive literature that accounts for competition [9], grazing [10], predation [11], facilitation [12], and the interplay between biotic and abiotic factors in structuring intertidal communities [13]. These processes are considered in environmental stress models that predict patterns of variation in biotic interactions and species diversity across environmental stress gradients [14]–[17].

In recent years, variability in intertidal community structure has been increasingly evaluated at different horizontal scales in terms of spatial resolution [4], [17]. Resolution may be defined as the size of the individual areas among which ecological properties (e.g. community structure) are compared. Examples are patch scale, often meaning in the literature areas between 10s of cm and a few m in width, and site scale, often meaning areas 10s of m in width. At fine resolutions (differences within nearby patches), causes of horizontal variability in community structure include physical disturbance [18], frond sweeping by canopy-forming seaweeds [19], substrate pre-emption [20], availability of refugia [3], and variations in grazing activity [21]. At broader resolutions (differences among shores, each one commonly meant to be between 100s and 1000s of m in width), variation in community structure has been related to changes in wave exposure and ice scour [22], and recruitment [3], [23]. Correlative evidence suggests that coastal geomorphology affects the distribution of primary producers at broad spatial scales [24], which is linked to broad-scale patterns in benthic community structure [25]. For regions harbouring the same basic biota, the highest variation in species abundance and community structure often occurs at fine resolutions [4].

Another approach to investigating scale effects in ecology is to focus on sampling interval, that is, the average distance between sampling units from which variation in ecological properties is calculated. Using this meaning of scale, it is thought that broad-scale processes may add extra variability to the patchiness commonly observed at fine scales on rocky shores [5]. Since many intertidal species have a planktonic stage, broad-scale processes are relevant to the structure of intertidal communities [15], [26], [27] and may cause a larger amount of variation than that resulting from the strong vertical stress gradient on rocky shores. However, so far only one study has contrasted vertical variation across intertidal elevations with horizontal variation measured along the shoreline at different sampling intervals [28]. Using intertidal systems from the NW Mediterranean coast, that study found that vertical variation in community structure was larger than horizontal variation measured at small scales but lower than horizontal variation measured at large scales. However, Benedetti-Cecchi [28] cautioned against considering such findings as universal for marine rocky shores, therefore calling for equivalent tests to be done on coasts representing different environments and biotas.

In this paper, we report the results of a study done using rocky intertidal systems from the cold-temperate shores of Helgoland Island, NE Atlantic. We tested the hypothesis that vertical variation in community structure is higher than fine-scale horizontal variation but lower than broad-scale horizontal variation. To test these predictions, we used a nested sampling design to quantify species abundances across the desired horizontal spatial scales in terms of differences in sampling interval. To help explain the observed community patterns, we also analysed the variability in the abundance of the species with the highest contribution to the spatial variation in community structure.

## Methods

### Study places

**Figure 1:**
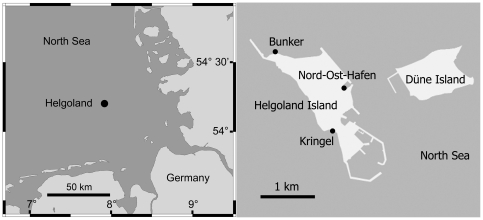
Location of the 3 studied shores on Helgoland Island, NE Atlantic.

We evaluated vertical and horizontal patterns in rocky intertidal community structure in July–August 2008 on 3 shores on Helgoland Island, which is located off the coast of mainland Germany, NE Atlantic (Fig. 1): Bunker (54° 11′ 32″ N, 7° 52′ 35″ E), Kringel (54°10′ 60″ N, 7° 53′ 15″ E), and Nord-Ost-Hafen (54° 11′ 00″ N, 7° 53′ 34″ E). Our research adheres to the legal requirements of the Schleswig-Holstein state act of 24 April 1981 (classification number 791-4-37) that declared Helgoland Island a nature reserve and allows ecologists to access sites to accomplish field research.

A nested sampling design was employed. On each of the 3 study shores, we randomly established 3 sites that were spaced 5–10 m from one another following the shoreline. Within each site, we randomly established 2 patches that were spaced a few m from one another. At each patch, we randomly deployed three 25 cm×25 cm quadrats that were spaced a few 10s of cm from one another at each of 3 intertidal elevations (low, middle, and high intertidal zones).

On each shore, we surveyed the full vertical range of elevation between 0 m and an upper intertidal boundary determined using ecological indicators. On marine shores, waves during high tides wash the substrate at higher elevations than the high-water level indicated by tide tables (which provide predictions for still-water conditions). Thus, the regular occurrence of wave action expands the vertical range where sessile organisms can live, so different elevation zones (high, middle, and low) are wider and higher as wave exposure increases [30]. Because of that, to compare community structure from areas with a given degree of emersion-related stresses occurring on shores differing in wave exposure, community measurements must be done on each shore at elevations that account for the local effects of waves as described above. The sessile, perennial species occurring highest on the shore under all wave exposures in a given region are excellent indicators of the upper intertidal boundary, because their upper distribution limit on different shores represents a summary of the local wave regime. As the upper intertidal boundary for our study shores, we used the upper distribution limit of the brown seaweed *Fucus spiralis*, because this is the sessile, perennial species occurring highest on the 3 shores. Once the upper boundary was determined on each shore, we divided the intertidal range in three zones of equal vertical extent (high, middle, and low zones). We took all elevation measurements to the nearest cm using a theodolite and a tide pole. All boundaries were permanently marked with screws bolted to the rocky substrate. The upper intertidal boundary was 2.3 m at Bunker, 1.8 m at Kringel, and 2.0 m at Nord-Ost-Hafen.

### Sampling

For each quadrat, we identified all seaweeds and invertebrates (>1 mm) using field guides [29]–[31] and taxonomic keys [32], [33]. Because of small size or morphological overlap with similar species, a few taxa were difficult to identify to the species level. Such organisms were classified to the lowest possible taxonomic level (usually genus), as normally done in field studies that identify all producers and consumers simultaneously in communities [34], [35].

For each quadrat, we measured the percent cover of each species using a 25 cm×25 cm frame divided in 100 equal sections with monofilament line. For data analyses, it was important to have abundance data expressed in the same measurement unit for every species. We chose percent cover to quantify abundance because alternative measures (e.g. density of individuals) cannot always be determined reliably for clonal organisms [36] or (e.g. biomass) would have implied destructive sampling of numerous shore areas. We first measured the cover of canopy species before carefully moving the canopy aside to sample the understorey species. Organisms were not removed from the quadrats. Cover values were obtained from the projection of three-dimensional structures to the plane of the sampling frame. Total percent cover for a quadrat could exceed 100% because of the multilayered structure of assemblages. When the cover was <1% for a given species, we recorded it as 0.5% [37].

### Data analysis

To reveal spatial patterns in community structure on the studied shores, we used canonical analysis on principal coordinates (CAP), [38]. Constrained multivariate methods such as CAP use an *a priori* hypothesis to produce an ordination plot, so they allow one to detect patterns that could be masked by overall dispersion in unconstrained methods such as multidimensional scaling. We generated CAP ordination plots for each shore on the basis of a matrix of factors (elevation, patch, and site) fitted to a matrix of Bray-Curtis dissimilarities. We assessed the significance of fits with 1000 permutations.

In nested designs, sampling effort (and thus statistical power) increases lower in the hierarchy of scales, which prevents a direct comparison of the magnitude of variation among the different scales though hierarchical analysis of variance [28]. Thus, to test our main hypothesis, we used an alternative type of analysis of variance described by Underwood and Chapman [39] and Benedetti-Cecchi [28], for which we obtained independent estimates of vertical and horizontal variation from different subsets of quadrats pooled at the appropriate spatial scales to keep a constant intensity of sampling across scales.

To measure vertical and horizontal variation in community structure, we used PERMANOVA based on Bray-Curtis distances between pairs of quadrats [40]. This multivariate technique tested for the effects of elevation (3 levels: high, middle, and low) on community structure using the abundance data for all species from a selected set of quadrats depending on the scale of interest. In PERMANOVA, the total multivariate variance (i.e. pseudo-variance) is partitioned into mean squares due to the factor of interest (elevation) and the residual or error mean squares [41]. We estimated the multivariate pseudo-variance component for elevation by equating the observed mean squares of the elevation factor to the expected mean squares derived from the linear model: (MS_elevations_ – MS_residual_)/n [42], where n was the number of replicate quadrats within each elevation. The MS_residual_ thus corresponded to the pseudo-variance component for horizontal variation. We applied PERMANOVA to measure horizontal variation at the different desired scales (different sampling intervals) in the way explained below.

To measure vertical variation in community structure across the three elevation zones and horizontal variation at the patch scale (variation among quadrats 10s of cm apart within patches), we randomly selected one site from each shore. We used one-way PERMANOVAs separately for each available patch to measure variation. Six patches were available for this analysis (3 shores ×1 site selected per shore ×2 available patches per site), yielding 6 independent estimates of vertical variation and 6 estimates of horizontal variation at the patch scale.

To measure horizontal variation at the site scale (variation among quadrats a few m apart within sites), we randomly selected another site from each shore. For each elevation zone within each site, we pooled the quadrats from both patches and, then, randomly divided the resulting 6 quadrats into 2 groups of 3 quadrats each. For each site, we then generated 2 data sets, each one including species abundance data from 3 quadrats from each elevation zone. We then applied one-way PERMANOVAs separately to each data set to generate 2 measures of horizontal variability for each site using the residual mean squares. In this case, horizontal variability measured variation within patches plus the variation between patches within sites. Six data sets were available for this analysis (3 shores ×1 site selected per shore ×2 modified sets of quadrats within each site), yielding 6 estimates of horizontal variation at the site scale.

To measure horizontal variation at the shore scale (variation among quadrats 100s to 1000s of m apart), we used the abundance data from the remaining site within each shore. First, we pooled the quadrats from each elevation zone across the 3 shores. Then, we randomly divided the resulting 18 quadrats into 6 groups of 3 quadrats each. We then randomly generated 6 data sets, each one including abundance data from 3 quadrats from each elevation zone. After applying one-way PERMANOVAs separately to those data sets, we obtained 6 measures of horizontal variation using the residual mean squares. In this case, horizontal variability measured variation within patches plus variation between patches within sites plus variation among shores.

We also calculated univariate variance components based on the abundance of the taxa contributing most to the spatial variation in community structure, for which we used the quadrat grouping procedure described above for the multivariate approach. For the univariate analyses, we used one-way ANOVAs to obtain estimators of variance. When negative variance components (either multivariate or univariate) were obtained for any scale, we set them to zero under the assumption that they were sample underestimates of small or zero variances [28], [43]. We calculated the contribution of each taxon to the spatial variation in community structure with Similarity Percentage (SIMPER) routines. In this procedure, we calculated Bray-Curtis dissimilarity values among replicate plots, between groups, and within groups in the entire dataset of species percent covers. The average between-group dissimilarities were then broken down into separate contributions from each taxon [44]. Species contributions were calculated separately for the factors elevation, patch, site, and shore.

We tested the hypothesis that vertical variation in community structure is larger than fine-scale horizontal variation but smaller that broad-scale horizontal variation by applying a one-way ANOVA to the multivariate pseudo-variance component data obtained as explained above for each scale. In a similar way, we tested the same hypothesis for the variance component data of the taxa with highest contributions to the spatial variation in community structure. We considered “type of variation” as a fixed factor with 4 levels: vertical variation and patch, site, and shore-scale horizontal variation. We confirmed the homoscedasticity assumption by running Levene's tests after graphical exploration of residuals vs. fitted values and log_10_(*y* + 1) or square-root transformation of the data. After significant differences among treatments were detected by the ANOVA, we ran Dunnett's tests to evaluate the significance of pairwise differences. We considered Dunnett's test appropriate for our goals because this test compares each group against a “control” group, which in this case corresponded to the vertical variation. SIMPER routines were conducted with PRIMER v6. We did all other statistical analyses and ordinations with *R* environment version 2.12.2 [45]. We used the vegan package to compute the CAP ordinations and PERMANOVAs, the stats package to compute the one-way ANOVAs, and the multcomp package to compute Dunnett's tests.

## Results

We identified a total of 62 taxa (29 seaweeds and 33 invertebrates) among the 3 studied shores. Four sessile taxa were present in at least 30% of the quadrats, exhibiting a mean percent cover higher than 20%: the turf-forming red alga *Mastocarpus stellatus,* canopy-forming brown algae of the genus *Fucus* (*F. spiralis*, *F. vesiculosus*, and *F. serratus*), crustose algae (*Hildenbrandia rubra*, *Ralfsia* sp., and *Phymatolithon* spp.), and the acorn barnacle *Semibalanus balanoides*. The most common grazers and carnivorous invertebrates were periwinkles of the genus *Littorina* (*L. obtusata*, *L. saxatilis*, and *L. littorea*) and the green crab *Carcinus maenas,* respectively. The complete list of species appears in a related publication that utilised the abundance data to investigate vertical trends in species diversity as predicted by an environmental stress model [46]. SIMPER routines identified 16 taxa that explained up to 90% of average Bray-Curtis between-group dissimilarities within each type of variability (Table 1). However, *F. spiralis*, *F. serratus* (both species hereafter analysed together in the univariate context as *Fucus* spp.), and *M. stellatus* explained over 50% of dissimilarities across all comparisons (Table 1).

**Table 1:** Breakdown of average Bray-Curtis dissimilarities (untransformed data) indicating the contributions of different taxa to 90% of the average between-group dissimilarity for each studied type of variation.

	Vertical			Patch (horizontal)			Site (horizontal)			Shore (horizontal)		
Species	*–δ*	*–δ*%	*Σ–δ*%	*–δ*	*–δ*%	*Σ–δ*%	*–δ*	*–δ*%	*Σ–δ*%	*–δ*	*–δ*%	*Σ–δ*%
*Mastocarpus stellatus*	16.7	23.0	23.0	45.4	21.9	21.9	45.4	21.8	21.8	15.3	21.3	21.3
*Fucus spiralis*	14.1	19.1	42.2	19.1	18.6	40.4	18.6	18.7	40.5	14.7	19.8	41.0
*Fucus serratus*	10.2	14.1	56.2	26.1	14.7	55.1	26.5	14.7	55.2	10.3	14.4	55.4
*Blidingia* spp.	4.4	5.8	62.0	6.2	5.9	61.0	6.3	5.9	61.1	4.1	5.6	61.0
*Fucus vesiculosus*	3.5	4.8	66.8	5.1	5.1	66.1	5.0	5.1	66.1	3.5	5.0	66.0
*Ulva* sp. 2	2.3	3.3	70.0	5.6	3.3	69.4	5.6	3.3	69.4	2.4	3.2	69.2
*Semibalanus balanoides*	2.2	3.0	73.0	4.8	3.1	72.5	4.8	3.1	72.5	2.3	3.1	72.4
*Chondrus crispus*	2.0	2.7	75.7	2.5	2.6	75.0	2.5	2.6	75.0	1.8	2.5	74.8
*Phymatolithon* spp.	1.9	2.6	78.3	1.9	2.8	77.8	1.8	2.8	77.8	2.1	3.0	77.8
*Ulva* sp. 1	1.8	2.5	80.8	4.4	2.5	80.3	4.4	2.5	80.3	1.8	2.5	80.3
*Flustrellidra hispida*	1.6	2.3	83.1	5.1	2.3	82.6	5.2	2.3	82.7	1.8	2.6	82.8
*Cladophora rupestris*	1.5	2.1	85.2	3.9	2.2	84.8	4.0	2.2	84.8	1.5	2.1	85.0
*Littorina littorea*	1.4	1.9	87.2	1.3	2.0	86.7	1.3	1.9	86.7	1.4	1.9	86.9
*Ralfsia* sp.	1.4	1.8	89.0	2.5	2.0	88.7	2.5	2.0	88.7	1.4	2.0	88.8
*Verruca stroemia*	0.7	1.0	90.0	2.1	1.1	89.7	2.2	1.1	89.8	0.8	1.1	89.9
*Hildenbrandia rubra*	0.7	0.9	91.0	0.5	1.0	90.8	0.5	1.0	90.8	0.7	1.0	90.9

***–δ*** =  average contribution of taxon *i* to between-group dissimilarities; ***–δ***% = average percentage contribution of taxon *i*; ***Σ–δ***% = cumulative average percentage contribution at the level of taxon *i.* The contribution of each taxon was averaged across all between-group pairwise comparisons.

**Figure 2:**
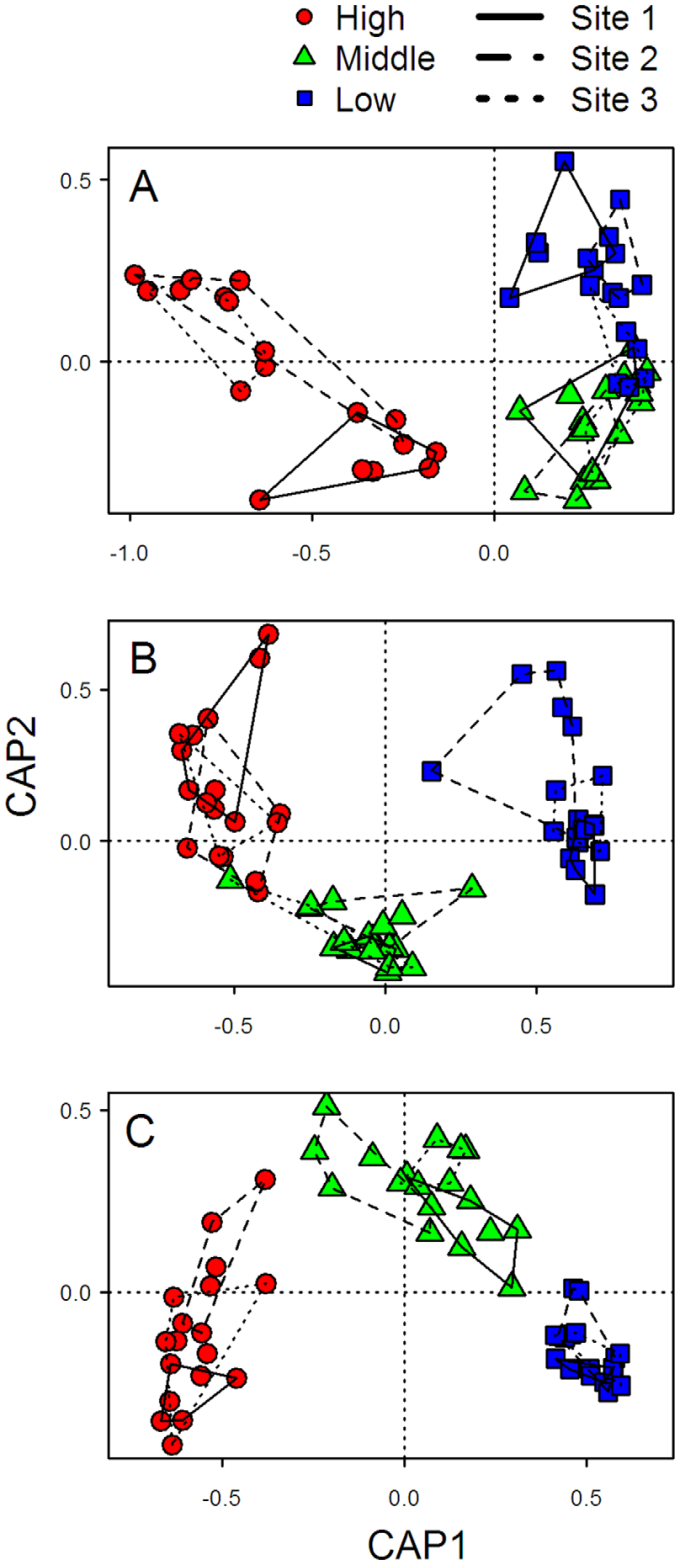
Canonical analysis of principal coordinates (CAP) ordinations showing the axes that best discriminate species assemblages at the 3 intertidal elevations surveyed on the 3 study shores: (A) Bunker, (B) Kringel, and (C) Nord-Ost-Hafen. The first and second canonical axes explained 63% and 14% of the total variation, respectively, in Bunker, 56% and 18% respectively in Kringel, and 62% and 16% respectively in Nord-Ost-Hafen.

The CAP ordinations revealed evident differences in community structure among the three intertidal elevations on the studied shores (Fig. 2). Fits for each shore were statistically significant (*P*<0.005). Differences among sites within shores were less marked, although they were indeed clear in some cases. For example, at the high intertidal zone of Bunker, quadrats from sites 1 and 3 were well separated in the multivariate space (Fig. 2). At Nord-Ost-Hafen, sites 1 and 2 showed separation at the high intertidal zone and, to a lesser degree, at the middle intertidal zone (Fig. 2).

**Figure 3:**
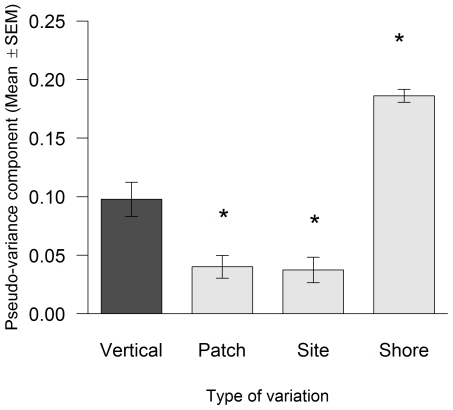
Vertical variation in community structure and horizontal variation in community structure for the 3 spatial scales measured in terms of sampling interval (mean ± SEM, n = 6). The asterisks over the bars for the horizontal scales indicate significant differences relative to vertical variation (Dunnett's tests, *P*<0.05).

Multivariate pseudo-variance components significantly differed among the studied types of spatial variation (Fig. 3; ANOVA on log_10_(*y* +1)-transformed data: *F*_3, 20_ = 39.53, *P*<0.001). Vertical variation in community structure was significantly higher than patch- and site-scale horizontal variation and significantly lower than shore-scale horizontal variation (Fig. 3; Dunnett's tests, *P*<0.05).

**Figure 4:**
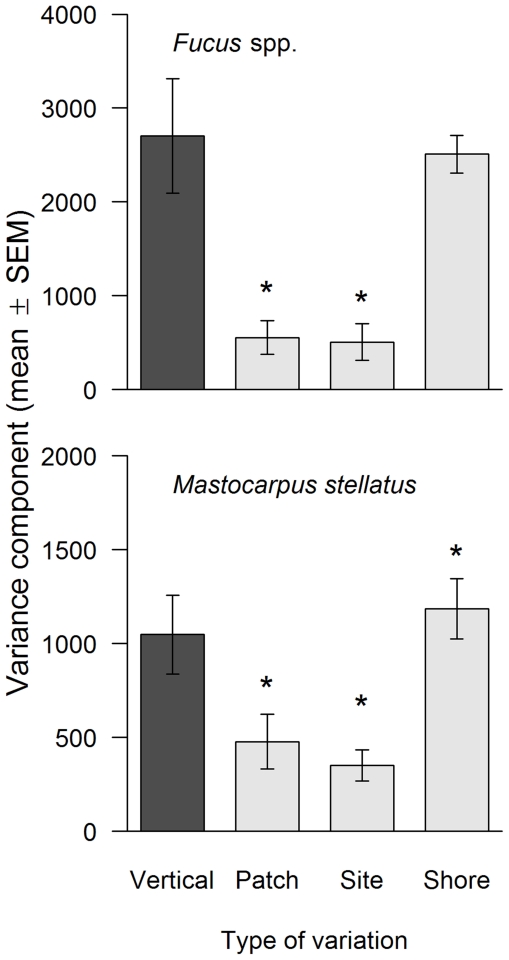
Vertical variation in the abundance of dominant taxa (*Fucus* spp. and *Mastocarpus stellatus*) and horizontal variation in such abundances for the 3 spatial scales measured in terms of sampling interval (mean ± SEM, n = 6). An asterisk over a bar for a horizontal scale indicates a significant difference relative to vertical variation (Dunnett's tests, *P*<0.05).

The type of spatial variation also had significant effects on variance components for *Fucus* spp. and *M. stellatus* (Fig. 4; ANOVA: *F*_3, 20_ = 14.23, *P*<0.001 for *Fucus* spp. and *F*_3, 20_ = 6.94, *P* = 0.002 for *M. stellatus*). For *Fucus* spp., the vertical variation in abundance was significantly higher than patch- and site-scale horizontal variation (Dunnett's tests, *P*<0.05) and statistically similar to shore-scale horizontal variation (Fig. 4; Dunnett's tests, *P*>0.05). For *M. stellatus*, the vertical variation in abundance was significantly higher than patch- and site-scale horizontal variation and significantly lower than shore-scale horizontal variation (Fig. 4; Dunnett's tests, *P*<0.05).

## Discussion

The pronounced vertical variation in community structure found on Helgoland shores corroborates previous findings for other intertidal systems [8], [47]. The observed vertical differences are likely primarily a result of the marked variation in environmental stress known to occur between low and high elevations [7]. Now, fine-scale horizontal variation in species assemblages is a common attribute of marine benthic habitats. The evidence comes from studies conducted in subtidal areas [48], [49], soft bottoms [50], coral reefs [51], and seagrass beds [52]. Yet, our results showed that vertical variation in community structure across elevations was higher than horizontal variation measured at fine spatial scales in terms of sampling interval (patches and sites). Conversely, vertical variation was lower than variation measured at our largest horizontal scale (shores). These findings suggest that broad-scale structuring processes along the coastline may be particularly relevant for intertidal community organization in Helgoland. In addition, broad-scale processes arise as being as important (or more) in determining community structure as the abiotic stresses that act so pervasively on marine rocky shores across the vertical elevation gradient [7].

The vertical gradient of abiotic stress that occurs in rocky intertidal habitats determines changes in the intensity of interspecific interactions that also influence vertical patterns in community structure [11], [20], [21]. Previous experimental work conducted in Helgoland suggested that herbivory by *Littorina littorea* and competition for space between mussels and *Fucus* spp. structured communities at middle elevations, while predation by *Carcinus maenas* shaped communities at low elevations [53]. More recently, however, the introduction to Helgoland shores of potentially dominant species, such as *Mastocarpus stellatus,* has been followed by a reduction in the abundance of mussels and an associated change in community structure [54]. Although new experimental work is therefore needed to understand what interspecific interactions are currently structuring intertidal communities in Helgoland, it is apparent that biological relationships still play a major role in determining species composition together with abiotic stresses across elevations.

It is now relevant to ask what factors might have determined the larger horizontal variation in community structure measured at the shore scale compared with vertical variation across elevations. On Helgoland Island, propagules are transported away from coastal waters [55], suggesting that propagule retention on local shores is probably low. Thus, limited connectivity among shores is a potential mechanism that may have led to the observed high horizontal variability across shores. This notion is in line with work conducted in intertidal systems from other oceanic islands (e.g. the Azores) [5] and in island coral reefs [56], where dispersal limitation leads to broad-scale variation in macrobenthic assemblages and fish populations, respectively. Scale-dependent effects derived from recruitment variability have been described for rocky shores [3], [57], indicating that studies on the spatial scales of propagule availability might help to explain the variation noted across shores in Helgoland.

Foundation species, such as *Fucus* spp. and *M. stellatus,* are known to have strong effects on the assembly, maintenance, and resilience of community structure [14], [58], [59]. Intertidal algal canopies can have positive effects on understory species by alleviating thermal and desiccation stress during low tides [60], [61], but also negative effects through pre-emption of space or the action of sweeping fronds [19], [62]. Moore et al. [63] suggested that broad-scale alterations in the distribution of fucoid seaweeds can have profound effects on community-level properties due to variation in shelter availability for benthic grazers. In addition, Bertocci et al. [59] showed that the consequences of removal of fucoid canopies for community structure depend on the presence or absence of additional destructive events. On the other hand, increased cover of *M. stellatus* negatively influences post-settlement mortality of *F. serratus* at Bunker [64], in a similar way as *Chondrus crispus*, morphologically similar to *M. stellatus*, affects recruitment of *F. evanescens* in eastern Canada [65]. On our study shores, both *M. stellatus* and *Fucus* spp. are often the most abundant taxa [48]. Our analyses indicated that *M. stellatus* showed the same spatial patterns of variability in abundance than the patterns of variation found for community structure, hinting for potential canopy effects of this bioengineer on many other species. *Fucus* spp. also showed a higher degree of variation in abundance at the shore scale than at the two finer horizontal scales, again suggesting that the dominant canopy-forming seaweeds may have strongly contributed to making a large broad-scale horizontal variation in community structure.

Physical attributes of marine shores may also affect broad-scale patterns in community variability. For example, differences in substrate topography and roughness may influence species composition through indirect effects on herbivore activity [66], [67], which can vary across spatial scales [21]. Although the 3 studied shores in Helgoland are rocky, granite substrate is the norm at Nord-Ost-Hafen, concrete substrate predominates with sand suspended in seawater due to a nearby beach at Kringel, and concrete substrate predominates over a matrix of sandstone at Bunker. Thus, experimentally evaluating the possible effects of substrate type on community structure could be a useful next step to understanding shore-scale effects in Helgoland. Physical stresses such as wave action are also known to influence community structure at the shore scale [47]. In Helgoland, *in situ* measurements using dynamometers have revealed that wave exposure is higher at Bunker than at Nord-Ost-Hafen and Kringel, being similar between these last two shores [46]. Thus, wave exposure might explain the horizontal variation at the shore scale to a certain degree. Experiments will also be needed to elucidate the role of wave action in generating shore-scale variation in intertidal species composition in Helgoland.

Our study conducted on cold-temperate NE Atlantic shores supports the previous findings of a study done on warm-temperate NW Mediterranean shores [28]. The fact that similar results were obtained from systems largely different in terms of physical (e.g. tidal range, sea surface temperature, productivity) and biotic (e.g. species composition) conditions could be interpreted as evidence that either (1) processes producing broad-scale variability operate similarly across different environmental conditions and species pools, or that (2) different processes may lead to similar patterns in spatial variability. The identity and functional characteristics of species strongly influence ecosystem functioning and seem to determine structural processes in natural communities [68]. Therefore, it is likely that different processes may have led to strikingly similar patterns, as demonstrated in comparative studies conducted across northern Atlantic coasts [69].

In summary, ours is the second study comparing ecological variation across a well known vertical gradient of environmental stress (the intertidal elevation gradient) with variation occurring at a variety of spatial scales (in terms of sampling interval) along the coastline. Our multivariate analyses have indicated that vertical variation in community structure was larger than horizontal variation at fine scales but smaller than that at broad scales. In turn, the univariate analyses on dominant taxa suggest that variation in the abundance of canopy-forming species may have contributed to generating the large degree of variation in community structure observed at the shore scale. In other words, broad-scale processes may add an important amount of variability to the pervasive small-scale patchiness observed along marine shores. Future studies on species distribution and community structure should benefit from applying a multi-scale approach in the face of increasing frequency and intensity of climate-change related impacts on natural communities and resources.
